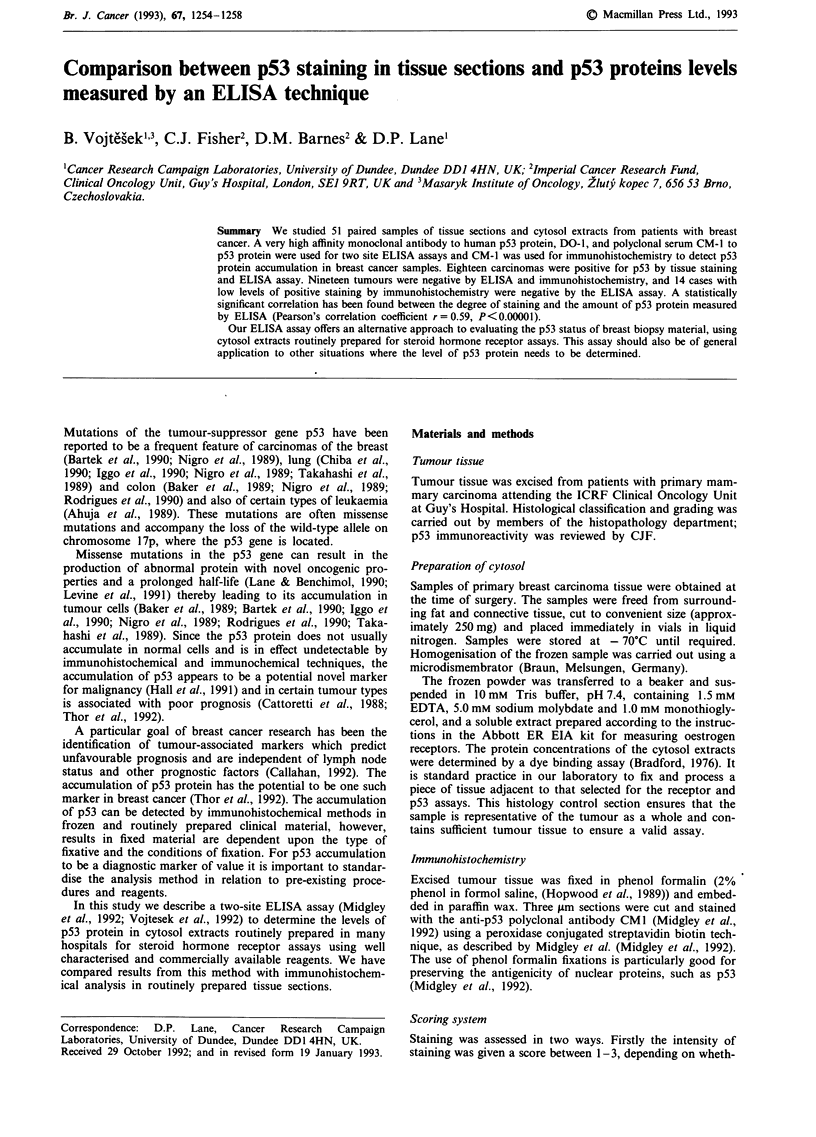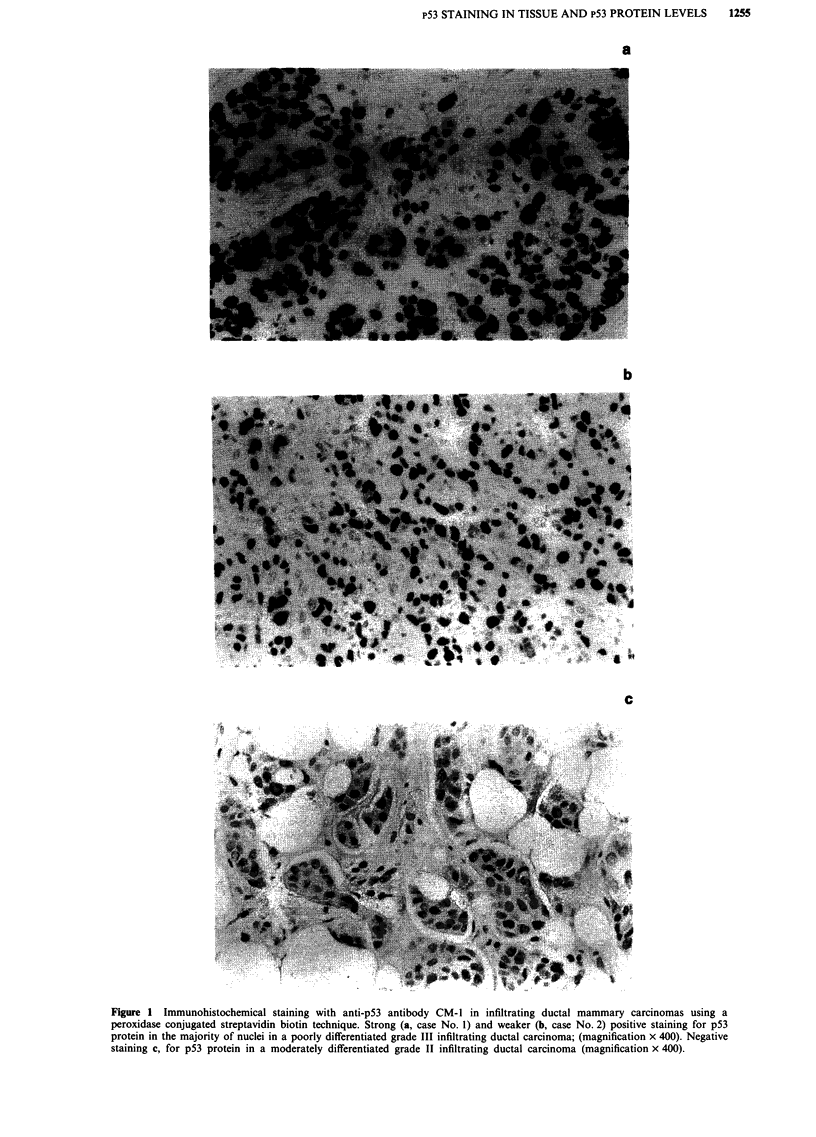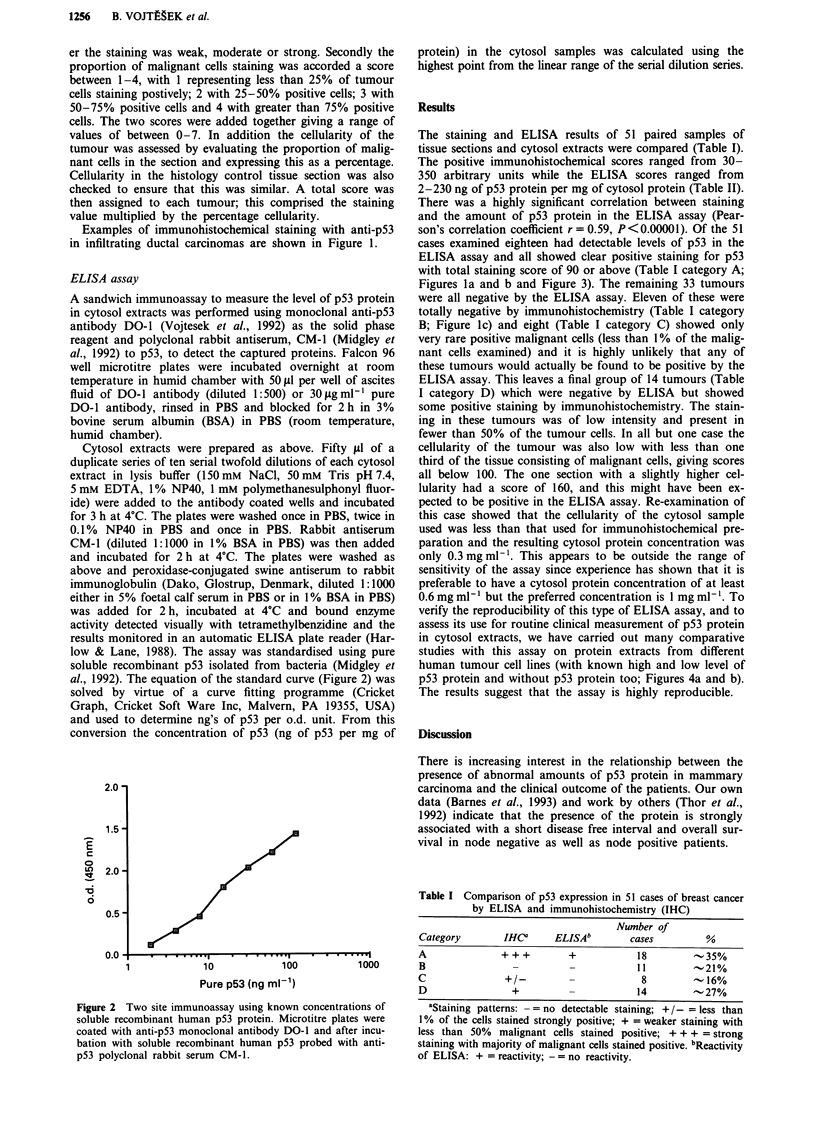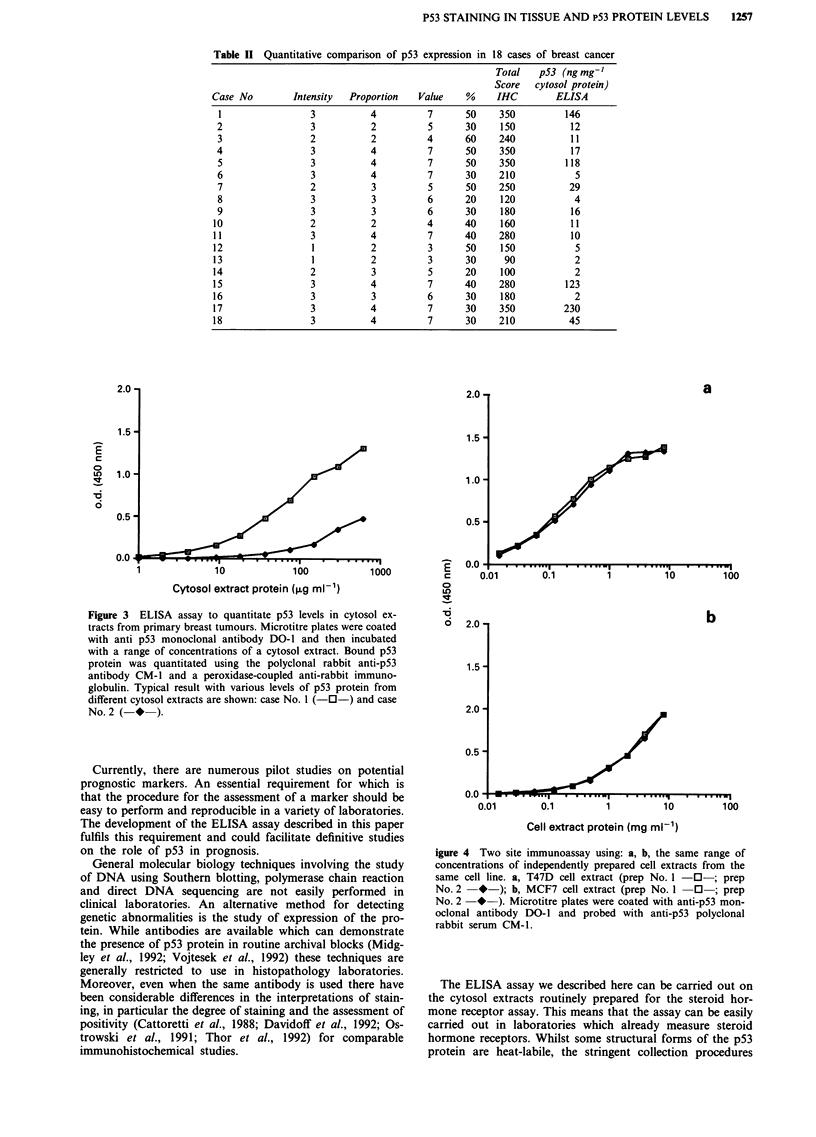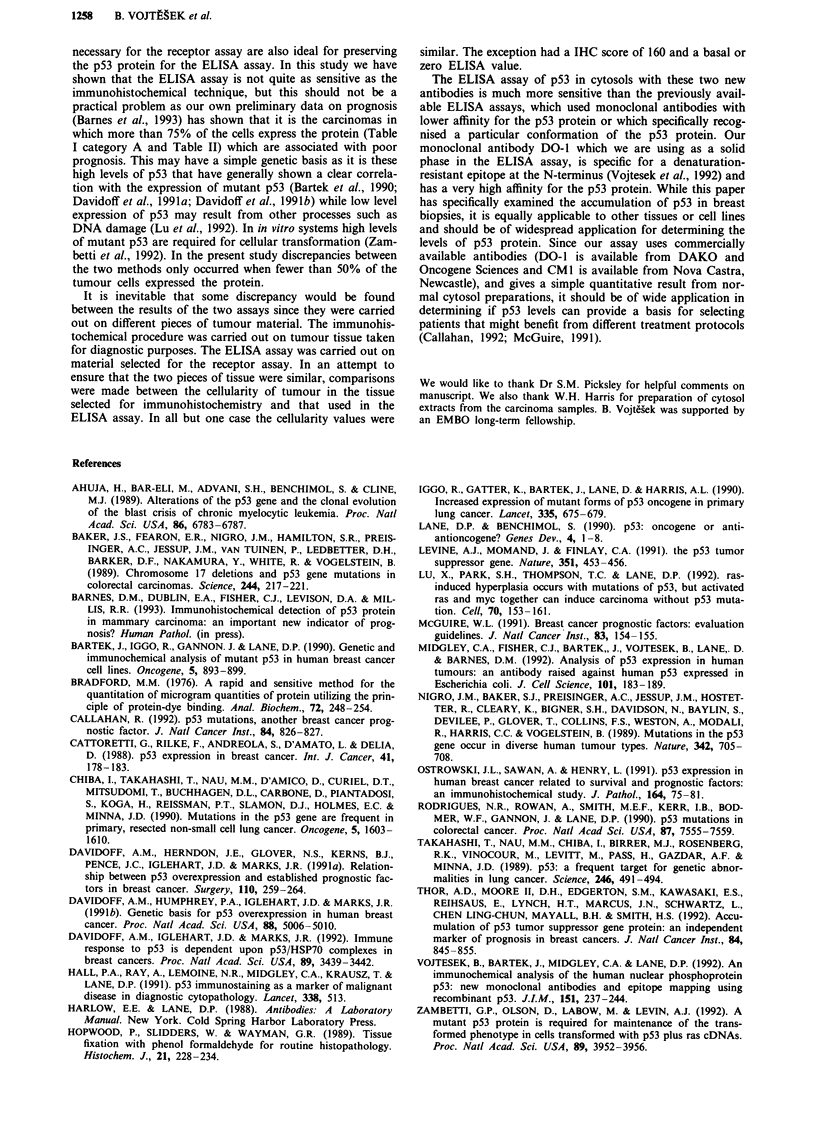# Comparison between p53 staining in tissue sections and p53 proteins levels measured by an ELISA technique.

**DOI:** 10.1038/bjc.1993.234

**Published:** 1993-06

**Authors:** B. Vojtĕsek, C. J. Fisher, D. M. Barnes, D. P. Lane

**Affiliations:** Cancer Research Campaign Laboratories, University of Dundee, UK.

## Abstract

**Images:**


Br. J. Cancer (1993), 67, 1254–1258 © Macmillan Press Ltd., 1993

### Comparison between p53 staining in tissue sections and p53 proteins levels measured by an ELISA technique

B. Vojtěšek[1,3], C.J. Fisher[2], D.M. Barnes[2] & D.P. Lane[1]

[1]*Cancer Research Campaign Laboratories, University of Dundee, Dundee DD1 4HN, UK;* [2]*Imperial Cancer Research Fund, Clinical Oncology Unit, Guy's Hospital, London, SE1 9RT, UK and* [3]*Masaryk Institute of Oncology, Žlutý kopec 7, 656 53 Brno, Czechoslovakia.*

**Summary** We studied 51 paired samples of tissue sections and cytosol extracts from patients with breast cancer. A very high affinity monoclonal antibody to human p53 protein, DO-1, and polyclonal serum CM-1 to p53 protein were used for two site ELISA assays and CM-1 was used for immunohistochemistry to detect p53 protein accumulation in breast cancer samples. Eighteen carcinomas were positive for p53 by tissue staining and ELISA assay. Nineteen tumours were negative by ELISA and immunohistochemistry, and 14 cases with low levels of positive staining by immunohistochemistry were negative by the ELISA assay. A statistically significant correlation has been found between the degree of staining and the amount of p53 protein measured by ELISA (Pearson's correlation coefficient $r = 0.59$, $P < 0.00001$).

Our ELISA assay offers an alternative approach to evaluating the p53 status of breast biopsy material, using cytosol extracts routinely prepared for steroid hormone receptor assays. This assay should also be of general application to other situations where the level of p53 protein needs to be determined.

Mutations of the tumour-suppressor gene p53 have been reported to be a frequent feature of carcinomas of the breast (Bartek *et al.*, 1990; Nigro *et al.*, 1989), lung (Chiba *et al.*, 1990; Iggo *et al.*, 1990; Nigro *et al.*, 1989; Takahashi *et al.*, 1989) and colon (Baker *et al.*, 1989; Nigro *et al.*, 1989; Rodrigues *et al.*, 1990) and also of certain types of leukaemia (Ahuja *et al.*, 1989). These mutations are often missense mutations and accompany the loss of the wild-type allele on chromosome 17p, where the p53 gene is located.

Missense mutations in the p53 gene can result in the production of abnormal protein with novel oncogenic properties and a prolonged half-life (Lane & Benchimol, 1990; Levine *et al.*, 1991) thereby leading to its accumulation in tumour cells (Baker *et al.*, 1989; Bartek *et al.*, 1990; Iggo *et al.*, 1990; Nigro *et al.*, 1989; Rodrigues *et al.*, 1990; Takahashi *et al.*, 1989). Since the p53 protein does not usually accumulate in normal cells and is in effect undetectable by immunohistochemical and immunochemical techniques, the accumulation of p53 appears to be a potential novel marker for malignancy (Hall *et al.*, 1991) and in certain tumour types is associated with poor prognosis (Cattoretti *et al.*, 1988; Thor *et al.*, 1992).

A particular goal of breast cancer research has been the identification of tumour-associated markers which predict unfavourable prognosis and are independent of lymph node status and other prognostic factors (Callahan, 1992). The accumulation of p53 protein has the potential to be one such marker in breast cancer (Thor *et al.*, 1992). The accumulation of p53 can be detected by immunohistochemical methods in frozen and routinely prepared clinical material, however, results in fixed material are dependent upon the type of fixative and the conditions of fixation. For p53 accumulation to be a diagnostic marker of value it is important to standardise the analysis method in relation to pre-existing procedures and reagents.

In this study we describe a two-site ELISA assay (Midgley *et al.*, 1992; Vojtesek *et al.*, 1992) to determine the levels of p53 protein in cytosol extracts routinely prepared in many hospitals for steroid hormone receptor assays using well characterised and commercially available reagents. We have compared results from this method with immunohistochemical analysis in routinely prepared tissue sections.

Correspondence: D.P. Lane, Cancer Research Campaign Laboratories, University of Dundee, Dundee DD1 4HN, UK.
Received 29 October 1992; and in revised form 19 January 1993.

### Materials and methods

### *Tumour tissue*

Tumour tissue was excised from patients with primary mammary carcinoma attending the ICRF Clinical Oncology Unit at Guy's Hospital. Histological classification and grading was carried out by members of the histopathology department; p53 immunoreactivity was reviewed by CJF.

### *Preparation of cytosol*

Samples of primary breast carcinoma tissue were obtained at the time of surgery. The samples were freed from surrounding fat and connective tissue, cut to convenient size (approximately 250 mg) and placed immediately in vials in liquid nitrogen. Samples were stored at − 70°C until required. Homogenisation of the frozen sample was carried out using a microdismembrator (Braun, Melsungen, Germany).

The frozen powder was transferred to a beaker and suspended in 10 mM Tris buffer, pH 7.4, containing 1.5 mM EDTA, 5.0 mM sodium molybdate and 1.0 mM monothioglycerol, and a soluble extract prepared according to the instructions in the Abbott ER EIA kit for measuring oestrogen receptors. The protein concentrations of the cytosol extracts were determined by a dye binding assay (Bradford, 1976). It is standard practice in our laboratory to fix and process a piece of tissue adjacent to that selected for the receptor and p53 assays. This histology control section ensures that the sample is representative of the tumour as a whole and contains sufficient tumour tissue to ensure a valid assay.

### *Immunohistochemistry*

Excised tumour tissue was fixed in phenol formalin (2% phenol in formol saline, (Hopwood *et al.*, 1989)) and embedded in paraffin wax. Three μm sections were cut and stained with the anti-p53 polyclonal antibody CM1 (Midgley *et al.*, 1992) using a peroxidase conjugated streptavidin biotin technique, as described by Midgley *et al.* (Midgley *et al.*, 1992). The use of phenol formalin fixations is particularly good for preserving the antigenicity of nuclear proteins, such as p53 (Midgley *et al.*, 1992).

### *Scoring system*

Staining was assessed in two ways. Firstly the intensity of staining was given a score between 1–3, depending on wheth-

**Figure 1** Immunohistochemical staining with anti-p53 antibody CM-1 in infiltrating ductal mammary carcinomas using a peroxidase conjugated streptavidin biotin technique. Strong (**a**, case No. 1) and weaker (**b**, case No. 2) positive staining for p53 protein in the majority of nuclei in a poorly differentiated grade III infiltrating ductal carcinoma; (magnification × 400). Negative staining **c**, for p53 protein in a moderately differentiated grade II infiltrating ductal carcinoma (magnification × 400).

er the staining was weak, moderate or strong. Secondly the proportion of malignant cells staining was accorded a score between 1–4, with 1 representing less than 25% of tumour cells staining postively; 2 with 25–50% positive cells; 3 with 50–75% positive cells and 4 with greater than 75% positive cells. The two scores were added together giving a range of values of between 0–7. In addition the cellularity of the tumour was assessed by evaluating the proportion of malignant cells in the section and expressing this as a percentage. Cellularity in the histology control tissue section was also checked to ensure that this was similar. A total score was then assigned to each tumour; this comprised the staining value multiplied by the percentage cellularity.

Examples of immunohistochemical staining with anti-p53 in infiltrating ductal carcinomas are shown in Figure 1.

### *ELISA assay*

A sandwich immunoassay to measure the level of p53 protein in cytosol extracts was performed using monoclonal anti-p53 antibody DO-1 (Vojtesek *et al.*, 1992) as the solid phase reagent and polyclonal rabbit antiserum, CM-1 (Midgley *et al.*, 1992) to p53, to detect the captured proteins. Falcon 96 well microtitre plates were incubated overnight at room temperature in humid chamber with 50 µl per well of ascites fluid of DO-1 antibody (diluted 1:500) or 30 µg ml$^{-1}$ pure DO-1 antibody, rinsed in PBS and blocked for 2 h in 3% bovine serum albumin (BSA) in PBS (room temperature, humid chamber).

Cytosol extracts were prepared as above. Fifty µl of a duplicate series of ten serial twofold dilutions of each cytosol extract in lysis buffer (150 mM NaCl, 50 mM Tris pH 7.4, 5 mM EDTA, 1% NP40, 1 mM polymethanesulphonyl fluoride) were added to the antibody coated wells and incubated for 3 h at 4°C. The plates were washed once in PBS, twice in 0.1% NP40 in PBS and once in PBS. Rabbit antiserum CM-1 (diluted 1:1000 in 1% BSA in PBS) was then added and incubated for 2 h at 4°C. The plates were washed as above and peroxidase-conjugated swine antiserum to rabbit immunoglobulin (Dako, Glostrup, Denmark, diluted 1:1000 either in 5% foetal calf serum in PBS or in 1% BSA in PBS) was added for 2 h, incubated at 4°C and bound enzyme activity detected visually with tetramethylbenzidine and the results monitored in an automatic ELISA plate reader (Harlow & Lane, 1988). The assay was standardised using pure soluble recombinant p53 isolated from bacteria (Midgley *et al.*, 1992). The equation of the standard curve (Figure 2) was solved by virtue of a curve fitting programme (Cricket Graph, Cricket Soft Ware Inc, Malvern, PA 19355, USA) and used to determine ng's of p53 per o.d. unit. From this conversion the concentration of p53 (ng of p53 per mg of protein) in the cytosol samples was calculated using the highest point from the linear range of the serial dilution series.

**Figure 2** Two site immunoassay using known concentrations of soluble recombinant human p53 protein. Microtitre plates were coated with anti-p53 monoclonal antibody DO-1 and after incubation with soluble recombinant human p53 probed with anti-p53 polyclonal rabbit serum CM-1.

### Results

The staining and ELISA results of 51 paired samples of tissue sections and cytosol extracts were compared (Table I). The positive immunohistochemical scores ranged from 30–350 arbitrary units while the ELISA scores ranged from 2–230 ng of p53 protein per mg of cytosol protein (Table II). There was a highly significant correlation between staining and the amount of p53 protein in the ELISA assay (Pearson's correlation coefficient $r = 0.59$, $P < 0.00001$). Of the 51 cases examined eighteen had detectable levels of p53 in the ELISA assay and all showed clear positive staining for p53 with total staining score of 90 or above (Table I category A; Figures 1a and b and Figure 3). The remaining 33 tumours were all negative by the ELISA assay. Eleven of these were totally negative by immunohistochemistry (Table I category B; Figure 1c) and eight (Table I category C) showed only very rare positive malignant cells (less than 1% of the malignant cells examined) and it is highly unlikely that any of these tumours would actually be found to be positive by the ELISA assay. This leaves a final group of 14 tumours (Table I category D) which were negative by ELISA but showed some positive staining by immunohistochemistry. The staining in these tumours was of low intensity and present in fewer than 50% of the tumour cells. In all but one case the cellularity of the tumour was also low with less than one third of the tissue consisting of malignant cells, giving scores all below 100. The one section with a slightly higher cellularity had a score of 160, and this might have been expected to be positive in the ELISA assay. Re-examination of this case showed that the cellularity of the cytosol sample used was less than that used for immunohistochemical preparation and the resulting cytosol protein concentration was only 0.3 mg ml$^{-1}$. This appears to be outside the range of sensitivity of the assay since experience has shown that it is preferable to have a cytosol protein concentration of at least 0.6 mg ml$^{-1}$ but the preferred concentration is 1 mg ml$^{-1}$. To verify the reproducibility of this type of ELISA assay, and to assess its use for routine clinical measurement of p53 protein in cytosol extracts, we have carried out many comparative studies with this assay on protein extracts from different human tumour cell lines (with known high and low level of p53 protein and without p53 protein too; Figures 4a and b). The results suggest that the assay is highly reproducible.

### Discussion

There is increasing interest in the relationship between the presence of abnormal amounts of p53 protein in mammary carcinoma and the clinical outcome of the patients. Our own data (Barnes *et al.*, 1993) and work by others (Thor *et al.*, 1992) indicate that the presence of the protein is strongly associated with a short disease free interval and overall survival in node negative as well as node positive patients.

**Table I** Comparison of p53 expression in 51 cases of breast cancer by ELISA and immunohistochemistry (IHC)

| *Category* | *IHC*[a] | *ELISA*[b] | *Number of cases* | *%* |
|---|---|---|---|---|
| A | + + + | + | 18 | ~35% |
| B | – | – | 11 | ~21% |
| C | +/– | – | 8 | ~16% |
| D | + | – | 14 | ~27% |

[a]Staining patterns: – = no detectable staining; +/– = less than 1% of the cells stained strongly positive; + = weaker staining with less than 50% malignant cells stained positive; + + + = strong staining with majority of malignant cells stained positive. [b]Reactivity of ELISA: + = reactivity; – = no reactivity.

**Table II** Quantitative comparison of p53 expression in 18 cases of breast cancer

| *Case No* | *Intensity* | *Proportion* | *Value* | *%* | *Total Score IHC* | *p53 (ng mg⁻¹ cytosol protein) ELISA* |
|---|---|---|---|---|---|---|
| 1 | 3 | 4 | 7 | 50 | 350 | 146 |
| 2 | 3 | 2 | 5 | 30 | 150 | 12 |
| 3 | 2 | 2 | 4 | 60 | 240 | 11 |
| 4 | 3 | 4 | 7 | 50 | 350 | 17 |
| 5 | 3 | 4 | 7 | 50 | 350 | 118 |
| 6 | 3 | 4 | 7 | 30 | 210 | 5 |
| 7 | 2 | 3 | 5 | 50 | 250 | 29 |
| 8 | 3 | 3 | 6 | 20 | 120 | 4 |
| 9 | 3 | 3 | 6 | 30 | 180 | 16 |
| 10 | 2 | 2 | 4 | 40 | 160 | 11 |
| 11 | 3 | 4 | 7 | 40 | 280 | 10 |
| 12 | 1 | 2 | 3 | 50 | 150 | 5 |
| 13 | 1 | 2 | 3 | 30 | 90 | 2 |
| 14 | 2 | 3 | 5 | 20 | 100 | 2 |
| 15 | 3 | 4 | 7 | 40 | 280 | 123 |
| 16 | 3 | 3 | 6 | 30 | 180 | 2 |
| 17 | 3 | 4 | 7 | 30 | 350 | 230 |
| 18 | 3 | 4 | 7 | 30 | 210 | 45 |

**Figure 3** ELISA assay to quantitate p53 levels in cytosol extracts from primary breast tumours. Microtitre plates were coated with anti p53 monoclonal antibody DO-1 and then incubated with a range of concentrations of a cytosol extract. Bound p53 protein was quantitated using the polyclonal rabbit anti-p53 antibody CM-1 and a peroxidase-coupled anti-rabbit immunoglobulin. Typical result with various levels of p53 protein from different cytosol extracts are shown: case No. 1 (—□—) and case No. 2 (—◆—).

**igure 4** Two site immunoassay using: **a**, **b**, the same range of concentrations of independently prepared cell extracts from the same cell line. **a**, T47D cell extract (prep No. 1 —□—; prep No. 2 —◆—); **b**, MCF7 cell extract (prep No. 1 —□—; prep No. 2 —◆—). Microtitre plates were coated with anti-p53 monoclonal antibody DO-1 and probed with anti-p53 polyclonal rabbit serum CM-1.

Currently, there are numerous pilot studies on potential prognostic markers. An essential requirement for which is that the procedure for the assessment of a marker should be easy to perform and reproducible in a variety of laboratories. The development of the ELISA assay described in this paper fulfils this requirement and could facilitate definitive studies on the role of p53 in prognosis.

General molecular biology techniques involving the study of DNA using Southern blotting, polymerase chain reaction and direct DNA sequencing are not easily performed in clinical laboratories. An alternative method for detecting genetic abnormalities is the study of expression of the protein. While antibodies are available which can demonstrate the presence of p53 protein in routine archival blocks (Midgley *et al.*, 1992; Vojtesek *et al.*, 1992) these techniques are generally restricted to use in histopathology laboratories. Moreover, even when the same antibody is used there have been considerable differences in the interpretations of staining, in particular the degree of staining and the assessment of positivity (Cattoretti *et al.*, 1988; Davidoff *et al.*, 1992; Ostrowski *et al.*, 1991; Thor *et al.*, 1992) for comparable immunohistochemical studies.

The ELISA assay we described here can be carried out on the cytosol extracts routinely prepared for the steroid hormone receptor assay. This means that the assay can be easily carried out in laboratories which already measure steroid hormone receptors. Whilst some structural forms of the p53 protein are heat-labile, the stringent collection procedures necessary for the receptor assay are also ideal for preserving the p53 protein for the ELISA assay. In this study we have shown that the ELISA assay is not quite as sensitive as the immunohistochemical technique, but this should not be a practical problem as our own preliminary data on prognosis (Barnes et al., 1993) has shown that it is the carcinomas in which more than 75% of the cells express the protein (Table I category A and Table II) which are associated with poor prognosis. This may have a simple genetic basis as it is these high levels of p53 that have generally shown a clear correlation with the expression of mutant p53 (Bartek et al., 1990; Davidoff et al., 1991a; Davidoff et al., 1991b) while low level expression of p53 may result from other processes such as DNA damage (Lu et al., 1992). In *in vitro* systems high levels of mutant p53 are required for cellular transformation (Zambetti et al., 1992). In the present study discrepancies between the two methods only occurred when fewer than 50% of the tumour cells expressed the protein.

It is inevitable that some discrepancy would be found between the results of the two assays since they were carried out on different pieces of tumour material. The immunohistochemical procedure was carried out on tumour tissue taken for diagnostic purposes. The ELISA assay was carried out on material selected for the receptor assay. In an attempt to ensure that the two pieces of tissue were similar, comparisons were made between the cellularity of tumour in the tissue selected for immunohistochemistry and that used in the ELISA assay. In all but one case the cellularity values were similar. The exception had a IHC score of 160 and a basal or zero ELISA value.

The ELISA assay of p53 in cytosols with these two new antibodies is much more sensitive than the previously available ELISA assays, which used monoclonal antibodies with lower affinity for the p53 protein or which specifically recognised a particular conformation of the p53 protein. Our monoclonal antibody DO-1 which we are using as a solid phase in the ELISA assay, is specific for a denaturation-resistant epitope at the N-terminus (Vojtesek et al., 1992) and has a very high affinity for the p53 protein. While this paper has specifically examined the accumulation of p53 in breast biopsies, it is equally applicable to other tissues or cell lines and should be of widespread application for determining the levels of p53 protein. Since our assay uses commercially available antibodies (DO-1 is available from DAKO and Oncogene Sciences and CM1 is available from Nova Castra, Newcastle), and gives a simple quantitative result from normal cytosol preparations, it should be of wide application in determining if p53 levels can provide a basis for selecting patients that might benefit from different treatment protocols (Callahan, 1992; McGuire, 1991).

We would like to thank Dr S.M. Picksley for helpful comments on manuscript. We also thank W.H. Harris for preparation of cytosol extracts from the carcinoma samples. B. Vojtěšek was supported by an EMBO long-term fellowship.

### References

AHUJA, H., BAR-ELI, M., ADVANI, S.H., BENCHIMOL, S. & CLINE, M.J. (1989). Alterations of the p53 gene and the clonal evolution of the blast crisis of chronic myelocytic leukemia. *Proc. Natl Acad. Sci. USA*, **86**, 6783–6787.

BAKER, J.S., FEARON, E.R., NIGRO, J.M., HAMILTON, S.R., PREISINGER, A.C., JESSUP, J.M., VAN TUINEN, P., LEDBETTER, D.H., BARKER, D.F., NAKAMURA, Y., WHITE, R. & VOGELSTEIN, B. (1989). Chromosome 17 deletions and p53 gene mutations in colorectal carcinomas. *Science*, **244**, 217–221.

BARNES, D.M., DUBLIN, E.A., FISHER, C.J., LEVISON, D.A. & MILLIS, R.R. (1993). Immunohistochemical detection of p53 protein in mammary carcinoma: an important new indicator of prognosis? *Human Pathol.* (in press).

BARTEK, J., IGGO, R., GANNON. J. & LANE, D.P. (1990). Genetic and immunochemical analysis of mutant p53 in human breast cancer cell lines. *Oncogene*, **5**, 893–899.

BRADFORD, M.M. (1976). A rapid and sensitive method for the quantitation of microgram quantities of protein utilizing the principle of protein-dye binding. *Anal. Biochem.*, **72**, 248–254.

CALLAHAN, R. (1992). p53 mutations, another breast cancer prognostic factor. *J. Natl Cancer Inst.*, **84**, 826–827.

CATTORETTI, G., RILKE, F., ANDREOLA, S., D'AMATO, L. & DELIA, D. (1988). p53 expression in breast cancer. *Int. J. Cancer*, **41**, 178–183.

CHIBA, I., TAKAHASHI, T., NAU, M.M., D'AMICO, D., CURIEL, D.T., MITSUDOMI, T., BUCHHAGEN, D.L., CARBONE, D., PIANTADOSI, S., KOGA, H., REISSMAN, P.T., SLAMON, D.J., HOLMES, E.C. & MINNA, J.D. (1990). Mutations in the p53 gene are frequent in primary, resected non-small cell lung cancer. *Oncogene*, **5**, 1603–1610.

DAVIDOFF, A.M., HERNDON, J.E., GLOVER, N.S., KERNS, B.J., PENCE, J.C., IGLEHART, J.D. & MARKS, J.R. (1991*a*). Relationship between p53 overexpression and established prognostic factors in breast cancer. *Surgery*, **110**, 259–264.

DAVIDOFF, A.M., HUMPHREY, P.A., IGLEHART, J.D. & MARKS, J.R. (1991*b*). Genetic basis for p53 overexpression in human breast cancer. *Proc. Natl Acad. Sci. USA*, **88**, 5006–5010.

DAVIDOFF, A.M., IGLEHART, J.D. & MARKS, J.R. (1992). Immune response to p53 is dependent upon p53/HSP70 complexes in breast cancers. *Proc. Natl Acad. Sci. USA*, **89**, 3439–3442.

HALL, P.A., RAY, A., LEMOINE, N.R., MIDGLEY, C.A., KRAUSZ, T. & LANE, D.P. (1991). p53 immunostaining as a marker of malignant disease in diagnostic cytopathology. *Lancet*, **338**, 513.

HARLOW, E.E. & LANE, D.P. (1988). *Antibodies: A Laboratory Manual*. New York. Cold Spring Harbor Laboratory Press.

HOPWOOD, P., SLIDDERS, W. & WAYMAN, G.R. (1989). Tissue fixation with phenol formaldehyde for routine histopathology. *Histochem. J.*, **21**, 228–234.

IGGO, R., GATTER, K., BARTEK, J., LANE, D. & HARRIS, A.L. (1990). Increased expression of mutant forms of p53 oncogene in primary lung cancer. *Lancet*, **335**, 675–679.

LANE, D.P. & BENCHIMOL, S. (1990). p53: oncogene or anti-antioncogene? *Genes Dev.*, **4**, 1–8.

LEVINE, A.J., MOMAND, J. & FINLAY, C.A. (1991). the p53 tumor suppressor gene. *Nature*, **351**, 453–456.

LU, X., PARK, S.H., THOMPSON, T.C. & LANE, D.P. (1992). ras-induced hyperplasia occurs with mutations of p53, but activated ras and myc together can induce carcinoma without p53 mutation. *Cell*, **70**, 153–161.

MCGUIRE, W.L. (1991). Breast cancer prognostic factors: evaluation guidelines. *J. Natl Cancer Inst.*, **83**, 154–155.

MIDGLEY, C.A., FISHER, C.J., BARTEK,, J., VOJTESEK, B., LANE,. D. & BARNES, D.M. (1992). Analysis of p53 expression in human tumours: an antibody raised against human p53 expressed in Escherichia coli. *J. Cell Science*, **101**, 183–189.

NIGRO, J.M., BAKER, S.J., PREISINGER, A.C., JESSUP, J.M., HOSTETTER, R., CLEARY, K., BIGNER, S.H., DAVIDSON, N., BAYLIN, S., DEVILEE, P., GLOVER, T., COLLINS, F.S., WESTON, A., MODALI, R., HARRIS, C.C. & VOGELSTEIN, B. (1989). Mutations in the p53 gene occur in diverse human tumour types. *Nature*, **342**, 705–708.

OSTROWSKI, J.L., SAWAN, A. & HENRY, L. (1991). p53 expression in human breast cancer related to survival and prognostic factors: an immunohistochemical study. *J. Pathol.*, **164**, 75–81.

RODRIGUES, N.R., ROWAN, A., SMITH, M.E.F., KERR, I.B., BODMER, W.F., GANNON, J. & LANE, D.P. (1990). p53 mutations in colorectal cancer. *Proc. Natl Acad Sci. USA*, **87**, 7555–7559.

TAKAHASHI, T., NAU, M.M., CHIBA, I., BIRRER, M.J., ROSENBERG, R.K., VINOCOUR, M., LEVITT, M., PASS, H., GAZDAR, A.F. & MINNA, J.D. (1989). p53: a frequent target for genetic abnormalities in lung cancer. *Science*, **246**, 491–494.

THOR, A.D., MOORE II, D.H., EDGERTON, S.M., KAWASAKI, E.S., REIHSAUS, E., LYNCH, H.T., MARCUS, J.N., SCHWARTZ, L., CHEN LING-CHUN, MAYALL, B.H. & SMITH, H.S. (1992). Accumulation of p53 tumor suppressor gene protein: an independent marker of prognosis in breast cancers. *J. Natl Cancer Inst.*, **84**, 845–855.

VOJTESEK, B., BARTEK, J., MIDGLEY, C.A. & LANE, D.P. (1992). An immunochemical analysis of the human nuclear phosphoprotein p53: new monoclonal antibodies and epitope mapping using recombinant p53. *J.I.M.*, **151**, 237–244.

ZAMBETTI, G.P., OLSON, D., LABOW, M. & LEVIN, A.J. (1992). A mutant p53 protein is required for maintenance of the transformed phenotype in cells transformed with p53 plus ras cDNAs. *Proc. Natl Acad. Sci. USA*, **89**, 3952–3956.